# The Lewis A phenotype is a restriction factor for Rotateq and Rotarix vaccine-take in Nicaraguan children

**DOI:** 10.1038/s41598-018-19718-y

**Published:** 2018-01-24

**Authors:** Filemón Bucardo, Johan Nordgren, Yaoska Reyes, Fredman Gonzalez, Sumit Sharma, Lennart Svensson

**Affiliations:** 1 Department of Microbiology, Faculty of Medical Science, National Autonomous University of Nicaragua, León (UNAN-León), León, Nicaragua; 2 Division of Molecular Virology, Department of Clinical and Experimental Medicine, Linköping University, 581 83 Linköping, Sweden; 3 Department of Medicine, Karolinska Institute, Stockholm, Sweden

## Abstract

Histo-blood group antigens (HBGAs) and the Lewis and secretor antigens are associated with susceptibility to rotavirus infection in a genotype-dependent manner. Nicaraguan children were prospectively enrolled in two cohorts vaccinated with either RotaTeq RV5 (n = 68) or Rotarix RV1 (n = 168). Lewis and secretor antigens were determined by saliva phenotyping and genotyping. Seroconversion was defined as a 4-fold increase in plasma IgA antibody titer 1 month after administration of the first dose of the vaccine. Regardless of the vaccine administered, significantly fewer of the children with Lewis A phenotype (0/14) seroconverted after receiving the first vaccine dose compared to 26% (45/175) of those with the Lewis B phenotype and 32% (15/47) of the Lewis negative individuals (*P* < 0.01). Furthermore, following administration of the RV1 vaccine, secretor-positive ABO blood group B children seroconverted to a significantly lesser extent (5%) compared to secretor-positive children with ABO blood groups A (26%) and O (27%) (*P* < 0.05). Other factors such as pre-vaccination titers, sex, breastfeeding, and calprotectin levels did not influence vaccine-take. Differences in HBGA expression appear to be a contributing factor in the discrepancy in vaccine-take and thus, in vaccine efficacy in different ethnic populations.

## Introduction

The monovalent (RV1) and pentavalent (RV5) rotavirus vaccines (GlaxoSmithKline and Merck, respectively) have been successful in reducing the incidence of severe gastroenteritis worldwide. However, the vaccine efficacy varies widely between high income countries and low- and middle-income countries (LMIC)^1^.Clinical trials in several countries in Sub-Saharan Africa have shown efficacies of RV1 and RV5 ranging from 39 to 67%^2–4^. Effectiveness studies conducted in Nicaragua following RV5 introduction demonstrated that vaccination was associated with a lower risk (58%) of severe rotavirus diarrhea despite the higher efficacy indicated by clinical trials in Latin America (85%)^5^. Inhibition of infant immune responses to rotavirus vaccine by trans-placental or naturally-acquired antibodies and poor immune responses due to either vitamin deficiencies, concomitant administration with oral poliovirus vaccine, high burden of other enteric infections, environmental enteropathy or differences in intestinal microbiome composition have been suggested as contributing factors in the modest vaccine effectiveness in these countries^6–11^; however, none have been identified consistently. Recently, the role of histo-blood group antigens (HBGAs) in rotavirus vaccine-take has emerged as an important issue, particularly in the context of susceptibility of different populations to various rotavirus strains^12^. The HBGA family, which includes the antigens responsible for the secretor, Lewis and ABO phenotypes, have been recognized as susceptibility factors for norovirus and rotavirus^13,14^.

Individuals with functional *fucosyltransferase*−2 (*FUT2*) and −3 (*FUT3*) express the Lewis-B (LeB) antigen, but those with a non-functional *FUT2* express only Lewis-A (LeA). Moreover, individuals with a non-functional *FUT3* gene express neither LeA nor LeB (LeA-B-). LeB is present at high frequencies in individuals of European descent, with LeA-B- being relatively rare (approximately 7%)^15–17^. In contrast, the LeA-B- phenotype can be present at frequencies reaching 40% in some Latin America and African populations^15,18–20^. The LeA phenotype, representing approximately 20% of individuals of European descent, is present at particularly low frequency in Latin America (approximately 5%)^13^.

To date, 37 different rotavirus P genotypes have been identified, with P[8] and P[4] genotypes remaining the globally dominant strains worldwide^21^, while P[6] is relatively more common in Sub-Saharan Africa. Both RV1 and RV5 contain rotavirus strains of genotype P[8], with RV5 also having P[5] genotype specificity^22,23^.

Children with non-secretor and LeA phenotype are highly resistant to natural infections with P[8] rotavirus strains; therefore, we hypothesized that the vaccine-take among LeA children vaccinated with P[8] rotavirus strains will be lower than that among LeB children. To test this hypothesis, we analyzed HBGAs and rotavirus-specific IgA antibody responses in Nicaraguan children eligible for rotavirus vaccination.

## Results

### Lewis A phenotype influences the vaccine-take of RV1 and RV5

In the RV1 cohort (n = 168), the Lewis phenotype distribution was 71% LeB, 23% LeA-B- and 6% LeA. Pre-vaccination IgA seropositivity rates were 58% (69/119) for LeB, 49% (19/39) for LeA-B- and 60% (6/10) for LeA. The seroconversion rates were 22% for LeB, 31% for LeA-B- and 0% for LeA (Table 1). Similarly, no significant increase in post- vaccination IgA titers was observed for LeA (GMT 90 vs. 96), while the titer increased significantly (*P* = 0.001) for LeB (Table 2).

**Table 1:** HBGAs as predictors of rotavirus IgA seroconversion post RV1 or RV5 vaccination among Nicaraguan children.

**Variable**	**RV1**			**RV5**			**Both vaccines**		
	**N**	**Seroconversion** ^**a**^ **n (%)**	**Odds Ratio (95% CI)**	**N**	**Seroconversion n (%)**	**Odds Ratio (95% CI)**	**N**	**Seroconversion n (%)**	**Odds Ratio (95% CI)**
**Lewis Phenotype**									
LeB	119	26 (22)	1.0	56	19 (34)	1.0	175	45 (26)	1.0
LeA-B-	39	12 (31)	1.60 (0.71, 3.56)	8	3(38)	1.17 (0.25, 5.42)	47	15(32)	1.35 (0.67, 2.73)
LeA	10	0 (0)	N/A	4	0 (0)	N/A	14	0 (0)	N/A
**Secretor Phenotype**									
Secretor	156	37 (24)	1.0	62	20 (32)	1.0	218	57 (26)	1.0
Non-secretor	12	1 (8)	0.29 (0.04, 2.34)	6	2 (33)	1.05 (0.18, 6.22)	18	3 (17)	0.57 (0.16, 2.02)
**Secretor Genotype (G428A)**									
SeSe	90	18 (20)	1.0	34	11 (32)	1.0	124	29 (23)	1.0
Sese^**428**^	66	19 (29)	1.5 (0.77, 3.40)	27	9 (33)	1 0.0 (0.36, 3.06)	93	28 (30)	1.4 (0.79, 2.60)
se^**428**^se^**428**^	12	1 (8)	0.3 (0.04, 3.00)	6	2 (33)	1 0.0 (0.16, 6.60)	18	3 (17)	0.7 (0.18, 2.42)
**Blood phenotype**									
O	103	26 (25)	1.0	49	15 (31)	1.0	152	41 (27)	1.0
A	42	10 (24)	0.93 (0.40, 2.14)	10	5 (50)	2.27 (0.57, 9.01)	52	15 (29)	1.1 (0.55, 2.21)
B	22	2 (9)	0.30 (0.07, 1.35)	9	2 (22)	0.65 (0.12, 3.49)	31	4 (13)	0.40 (0.13, 1.22)
AB	1	0 (0)	N/A	0	0 (0)	N/A	1	0 (0)	N/A

^a^Seroconversion was defined as a four-fold increase in rotavirus specific IgA titers 28 days post-vaccination. Abbreviations: RV1 = monovalent rotavirus vaccine; RV5 = pentavalent rotavirus vaccine; CI = confidence interval, N/A = not apply.

**Table 2:** Association of HBGA phenotypes with pre- and post-vaccination IgA titers in RV1 or RV5 vaccinated Nicaraguan children.

**HBGAs phenotype**	**RV1 (n = 168)**					**RV5 (n = 68)**				
	**n (%)**	**Pre Vaccination IgA GMT (Q1 - Q3)** ^**a**^	**Post Vaccination IgA GMT (Q1 - Q3)**	**Fold-rise in GMT**	***p*** **value** ^**b**^	**n (%)**	**Pre Vaccination IgA GMT (Q1 - Q3)**	**Post Vaccination IgA GMT (Q1 - Q3)**	**Fold-rise in GMT**	***p*** **value** ^**b**^
**Lewis**										
LeB	119 (71)	90 (20–320)	130 (40–640)	1.4	0.001	56 (82)	70 (25–175)	156 (50–400)	2.2	<0.001
LeA-B-	39 (23)	85 (20–320)	119 (40–320)	1.4	0.182	8 (12)	42 (25–50)	154 (31–1300)	3.7	0.041
LeA	10 (6)	90 (20–460)	96 (35–400)	1.1	0.892	4 (6)	84 (44–175)	100 (50–400)	1.2	0.593
**Secretor**										
Secretor	156 (93)	90 (20–320)	128 (40–400)	1.4	<0.001	62 (91)	66 (25–100)	148 (50–400)	2.2	<0.001
Non-secretor	12 (7)	75 (20–350)	95 (40–380)	1.3	0.611	6 (9)	71 (44–125)	200 (50–700)	2.8	0.138
**Blood**										
O	103 (61)	100 (20–320)	149 (40–640)	1.5	<0.001	49 (72)	70 (50–100)	157 (50–400)	2.2	<0.001
A	42 (25)	74 (20–200)	101 (20–320)	1.4	0.411	10 (15)	62 (25–125)	162 (50–800)	2.6	0.116
B	22 (13)	74 (18–460)	87 (20–400)	1.2	0.866	9 (13)	54 (25–150)	117 (38–400)	2.1	0.027
AB	1 (0.6)	80 (80–80)	80 (80–80)	1	N/A	0	N/A	N/A	N/A	N/A
**Overall**	168	89 (20–320)	125 (40–400)	1.4	<0.001	68	67 (25–100)	152 (50–400)	2.3	<0.001

^a^Q1 and Q3 stand for Interquartile 25th and 75th, respectively.

^b^Wilcoxon Signed Ranks Test was used to compare change in rotavirus specific IgA following vaccination. Abbreviations: RV1 = monovalent rotavirus vaccine; RV5 = pentavalent rotavirus vaccine; GMT = geometric mean titer.

The Lewis phenotype distribution in the RV5 cohort (n = 68) was 82% LeB, 12% Le-A-B- and 6% LeA. Pre-vaccination IgA seropositivity rates were 41% (23/56) for LeB, 12% (1/8) for LeA-B-, and 75% (3/4) for LeA. In the RV5 cohort, children with the LeA phenotype did not seroconvert following vaccination (Table 1), nor did the IgA GMT increase significantly post-vaccination (84 vs. 100), but the number of LeA were too few to draw any reliable conclusion (Table 2). In contrast, rotavirus-specific IgA titers increased by 2.2- and 3.7-fold for LeB (*P* *<* 0.001) and LeA-B- (*P* = 0.059), respectively (Table 2).

Lewis genotyping (n = 28) of *FUT3* confirmed the phenotyping, with 17 (89%) of 19 LeA-B- and all nine Lewis-positives (1 LeA and 8 LeB) presenting the combination of SNPs (haplotypes) that define these phenotypes. Two Lewis-negative samples could not be verified genetically based on the five investigated SNPs.

### RV1 vaccination of non-secretor children results in a lower rate and extent of seroconversion

In the RV1 cohort, the distribution of secretor and non-secretor phenotypes was 93% and 7%, respectively, and all were confirmed by genotyping. Thus, all non-secretors were homozygous for the G428A mutation in *FUT2* (Table 1). Pre-vaccination, IgA seropositive rates for secretor and non-secretor phenotypes were 56% (87/156) and 58% (7/12), respectively. A lower rate of IgA seroconversion was observed in RV1 vaccinated non-secretor children (8%) compared with that of secretors (24%), (OR = 0.29, 95% CI: 0.04–2.3) (Table 1). Furthermore, there was a significant increase in IgA titers post-vaccination in secretors but not in non-secretors (*P* < 0.001) (Table 2).

In the RV5 cohort, the distribution of secretor and non-secretor phenotypes was 91% and 9%, respectively. The pre-vaccination IgA seropositivity rates for secretors and non-secretors were 39% (24/62) and 50% (3/6), respectively. In the RV5 cohort, similar rates of IgA seroconversion were observed in secretors (32%) and non-secretors (33%) (Table 1), and titers increased significantly post-vaccination for secretors (Table 2); however, the number of non-secretors was too low to draw any conclusion.

Importantly, all three seroconverted non-secretors in both vaccine cohorts were LeA-B- (Table 1). Overall, the fold-increase in GMT was higher in the RV5 cohort than that in the RV1 cohort (2.3 vs. 1.4), although both vaccines induced a significant increase in IgA titers (Table 2).

### ABO blood group B phenotype influences RV1 vaccine-take

In the RV1 cohort, the O, A, B and AB phenotype distribution was 61% for O, 25% for A, 13% for B and 0.6% for AB. For blood phenotypes O, A, B and AB, RV1-specific IgA seropositivity rates pre-vaccination were 61% (63/103), 48% (20/42), 45% (10/22) and 100% (1/1), respectively. While IgA seroconversion rates were similar in children with blood phenotypes O (25%) and A (24%), a remarkably lower rate of IgA seroconversion was observed in type B children (9%) compared with type O (OR = 0.3, 95% CI: 0.07–1.35) (Table 1). Similarly, type B children had the lowest rise in IgA GMT post-vaccination, while a significant increase was observed in type O children (Table 2). After stratifying blood phenotypes by secretor status, only 5% (1/19) of the type B secretors seroconverted (OR = 0.15, *P* *<* 0.05) compared with 26% (10/38) and 27% (26/98) of type A and type O secretors, respectively.

In the RV5 cohort, the O, A, B and AB phenotype distribution was 72%, 15%, 3% and 0%, respectively (Table 1). Pre-vaccination IgA seropositivity rates were 41% (20/49), 40% (4/10), 33% (3/9) for types O, A and B respectively. A modest proportion (22%) of type B children seroconverted, although the total number was low (Table 1). The highest rates of IgA seroconversion were observed in type A (50%) and type O (31%) children. Moreover, IgA titers increased significantly in type O children (*P* < 0.001), but not in type A (Table 2). There was a significant increase in IgA titers in type B children vaccinated with RV5 (*P* = 0.014) (Table 2). Similar results were observed after stratifying blood phenotypes by secretor status.

We then investigated a set of factors with the potential to interfere with vaccine-take among LeA and type B individuals (Table 3). Exclusive breast-feeding, sex and calprotectin levels were not negatively associated with seroconversion in either cohort. There was, however, a tendency for higher seroconversion rates in RV5 vaccinated children of mothers with IgA titers ≥320 in breast-milk (Table 3).

**Table 3:** Association between non-genetic characteristics and IgA seroconversion among vaccinated Nicaraguan children.

**Variables**	**RV1 (n = 168)**		**RV5 (n = 68)**		**Both Vaccines (n = 236)**		**p-value** ^**b**^
	No. enrolled children	Seroconversion^a^n (%)	No. enrolled children	Seroconversion n (%)	No. enrolled children	Seroconversion n (%)	
**Sex**							
Female	88	21 (24)	27	10 (37)	115	31 (27)	0.60
Male	80	17 (21)	41	12 (29)	121	29 (24)	
**IgA status pre-vaccination**							
Seropositive^c^	94	18 (19)	27	9 (33)	121	27 (22)	0.26
Seronegative	74	20 (27)	41	13 (32)	115	33(29)	
**Breast feeding**							
Non-Exclusive	89	18 (20)	53	18 (34)	142	36 (25)	0.93
Exclusive	79	20 (25)	10	3 (30)	89	23 (26)	
**IgA titers in breast-milk** ^**d**^							
≤ 160	28	5 (18)	42	10 (24)	70	15 (21)	0.411
320–639	11	2 (18)	15	7 (47)^e^	26	9 (35)	
640 ≤	19	3 (16)	10	4 (40)^f^	29	7 (24)	
**Calprotectin (ug/ml)** ^**g**^							
2.3 to 3.8 (25th)	7	1 (14)	16	6 (38)	23	7 (30)	0.53
3.9 to 4.7 (50th)	18	1 (6)	29	8 (28)	47	9 (19)	
4.8 to 9.7 (75th)	7	0 (0)	15	6 (40)	22	6 (27)	

^a^Seroconversion defined as a four-fold increase in serum IgA titers 28 days post-vaccination with RV1 or RV5.

^b^Chi-square test of equal proportions for both vaccines combined

^c^Seropositivity was defined as a titer of IgA ≥80 or ≥100 in RV1 or RV5, respectively.

^d^A subset of 58 RV1 and 67 RV5 mothers provided breast milk for IgA analysis. Titer categories were arbitrary defined.

^e^OR = 2.8, CI: 0.81–9.66, after comparing ≤160 vs 320–639 categories, respectively

^f^OR = 2.1, 0.50–9.10, after comparing ≤160 vs 640≤ categories, respectively

^g^A subset of 32 RV1 and 60 RV5 children provided stool samples for calprotectin analysis. Stratification of calprotectin concentration represent percentiles.

Abbreviations: RV1 = monovalent rotavirus vaccine; RV5 = pentavalent rotavirus vacci.

## Discussion

In this study, we investigated the influence of HBGA phenotypes on vaccine-take based on IgA levels and seroconversion rates in plasma after the first dose in two cohorts of children vaccinated with RV1 or RV5. The fact that natural infections with rotavirus occur early in life in Nicargua^24^, significantly increases the risk for natural rotavirus infection during the course of vaccination and could mask the correct serological response of the vaccine. By determining the serological response after the first does, we significantly limit that risk.

The seroconversion rates after one dose of RV1 and RV5 was 23% and 32%, respectively. This shows the importance of more than one dose to induce immunization at population level, and is similar to rates reported elsewhere^25^, but lower than other studies^26^. The modest seroconversion probably reflect that immune response was determined after only one dose. A direct comparison is often difficult since most studies report seroconversion rates after full course of vaccination.

None of the children with the LeA phenotype seroconverted in either of the cohorts, although the small number of LeA phenotype children in the RV5 cohort warrants cautious interpretation of these results. Previous studies have shown that individuals with the LeA and non-secretor phenotype are less susceptible to natural infection with P[8] rotavirus strains^12,27–30^. However, several of these studies have only investigated secretor geno/phenotypes and not Lewis status, thus there is still a lack of data regarding Lewis A and susceptibility to rotavirus of different genotypes. Furthermore, *in vitro* studies have demonstrated that P[8] rotavirus does not bind to LeA but to secretor antigens, such as H type 1 and LeB^31–33^. These observations suggest that, compared with LeA individuals, LeB individuals will develop a more robust immune response towards RV1 and RV5.

Furthermore, seroconversion rates among non-secretors in the RV1 cohort were lower than those among secretors. Moreover, all three non-secretors that did seroconvert were LeA-B-. The non-secretor and LeA-B- phenotype is globally extremely rare, and its effect on vaccine-take and/or natural susceptibility warrants further studies with larger sample sizes. The *FUT2* genotyping (G428A) yielded 100% correlation with phenotyping. Heterozygosity or homozygosity of the secretor genotype was not found to influence vaccine-take, which is in accordance with reports of natural infections^27^.

In this study, we further observed that the seroconversion rate was significantly lower in secretor phenotype children with blood type B compared to those with types O and A. A previous *in vitro* study showed that P[8] binding to type B saliva was significantly lower than that to types A/AB and O, suggesting that the type B epitope interferes with the binding by masking the H or LeB epitope^32^. The effect of blood type AB could not be assessed here due to the low prevalence (n = 1); blood type AB being rare in Latin America. Another study showed that the VP8* fragment of a P[8] strain had low binding activity to saliva from type B individuals as compared with O and A types^33^. Thus, our *in vivo* observation is in accordance with these *in vitro* studies. Furthermore, a similar finding was recently reported from Pakistan, where secretors with blood type O were more likely to seroconvert compared to non-blood type O individuals^25^, the majority of which were blood type B. Moreover, a recent study from Egypt found that rotavirus positive cases of gastroenteritis were significantly less prevalent in children with blood type B as compared with type A^34^. To our knowledge, the potential of the blood type B phenotype to reduce susceptibility to natural infection with P[8] strains has not yet been reported and further studies are warranted.

Details of the influence of pre-vaccination IgA titers on rotavirus vaccine-take are limited. It can be hypothesized that pre-vaccination immune responses might provide a booster effect, while pre-vaccine intestinal IgA might neutralize vaccine strains^35^. In the current study, pre-vaccination rotavirus seropositivity was not observed to significantly influence vaccine-take, suggesting that the vaccines are neither boosted nor neutralized by pre-vaccination rotavirus-specific IgA.

The high pre-vaccine IgA seropositivity rate (56%) found in the RV1 cohort (2015–2016) is in agreement with a birth cohort (n = 236 children) carried out in the same setting between 1991 and 1994, in that study >50% of the infants had evidence of past rotavirus infection by the age of 2 months^24^. The seropositivity rate (40%) in the RV5 cohort (2013–2014) is comparable with a previous report (30%) from the same setting and within the same time frame (September to November 2014)^36^. The differences of rotavirus seropositivity between the RV1 and RV5 cohorts may be associated with seasonal variation of natural rotavirus infections or increased transmission of RV1 strains. The observation that pre-vaccination IgA seropositivity rates were high across all HBGAs in the RV1 cohort suggest that there had been no difference in susceptibility to natural infections early in life or transmission of several rotavirus genotypes infecting children of different HBGAs. P[6] strains have been observed to readily infect non-secretors^12^, however this genotype is relatively rare in symptomatic children from Nicaragua^37^. Other putative reasons include asymptomatic neonatal infections, which are not routinely investigated and often caused by strains not circulating in older children^38^. Indeed, P[6] strains have been isolated from asymptomatic neonates from South Africa, Venezuela, Australia, Brazil, Sweden and India^38–41^. Furthermore, expression of HBGAs may also be developmentally regulated and different in children <1–2 month of age^42^, and the observed genetic susceptibility may not be absolute, particularly in the very young.

The observed associations between HBGA phenotype and vaccine-take were stronger in the RV1 cohort than those in the RV5 cohort, possibly due to the smaller sample size in the RV5 group, reducing the statistical power. However, we observed more robust increases in GMT in the RV5 cohort compared to those in the RV1 cohort for all HBGA phenotypes, indicating that RV5 might be more immunogenic after one dose. RV5 is a more complex vaccine, containing four human-bovine reassortant strains with the bovine P[5] genotype and one bovine strain with the human P-genotype P[8]. Whether the P[5] genotype rotavirus naturally infects humans or whether HBGA factors influence RV5 immunogenicity with respect to P[5] remains to be determined.

None of the investigated non-genetic factors, such as breastfeeding, calprotectin and IgA status pre-vaccination were found to influence seroconversion rates. An association between these variables and rotavirus vaccine-take has been reported previously^9,36,43,44^; however, the findings in different settings and populations are inconsistent, and the extent of the influence of these variables remains to be established.

A limitation of the study is the relatively low frequency of non-secretors and LeA phenotypes, which is the case for several Latin American populations^13,30^. This naturally limits analytical power regarding these phenotypes. However, nil of 14 children seroconverted which suggests an association between Lewis A and lack of vaccine-take after first dose. Extrapolating from the results, showing less seroconvertion in Lewis A and bloodgroup B children, it can be speculated that the high prevalence of secretors, LeB, and O and A blood phenotypes at the population level might induce a better immune response to the vaccine available in Latin America compared to that in other populations having higher prevalence of Lewis A and/or blood group B.

It is important to note that LeA and non-secretor children who are resistant to the live-attenuated vaccines would also be resistant to naturally occurring strains of that genotype, and would therefore, not appear in vaccine failure data. Thus, in a P[8] environment, such as Latin America as well as Europe and North America, lower vaccine take in some children would be masked by their resistance to wild circulating strains. P[6] is the only P-genotype that has been shown to readily infect non-secretors in authentic rotavirus infections^12^. This genotype is globally rare, but present at high frequencies in some populations in sub-Saharan Africa. Thus, in these diverse P-genotype environments, the vaccine failures will be apparent and vaccine efficacy expected to be lower, as has been described from several African countries.

## Methods

### Subjects

A total of 236 Nicaraguan infants (aged approximately 8 weeks) eligible for rotavirus vaccination were prospectively enrolled at household or community clinics between July 2013 and November 2016. Rotavirus-specific plasma IgA titers were determined pre- and 28 days post-administration of the first dose of RV1 (n = 168) or RV5 (n = 68). Evaluation of seroconversion following all doses of vaccination was not considered in order to limit the effect of the very early natural rotavirus infections previously observed in Nicaragua^24^. A subset of 125 mothers of children in the RV1 (n = 58) and RV5 (n = 76) cohorts provided breast-milk for rotavirus-specific IgA analysis. The protocol and questionnaire used in this study were reviewed and approved by the Ethical committee for Biomedical Research of UNAN-León (Acta No. 18, 2012) and the methods performed in accordance with guidelines and regulations. Written informed consent was obtained from the parents of all children included in this study.

### Sampling

Saliva and blood samples were collected by a pediatric nurse. Saliva collected using a sterile cotton-swab was placed into 500 µl phosphate buffered saline (PBS, pH 7.2). Blood (approximately 2 ml) and breast-milk (approximately 5 ml) samples were collected into EDTA-coated glass tubes and plastic containers, respectively, labeled with the child’s code and date of collection. Samples were transported at 4 °C to the Department of Microbiology, UNAN-León where ABO blood phenotyping was performed on the day of collection, while saliva and breast-milk samples were stored at −20 °C. Following centrifugation of blood (5,000 rpm for 5 min), plasma was collected and stored for rotavirus-specific IgA titer analysis and the buffy coat was stored at 4 °C prior to DNA purification for *FUT2* and *FUT3* genotyping.

### Collection of epidemiological information

Clinical information such as age, sex, weight, height, feeding-habits (including breastfeeding) and history of previous diarrheal episodes was collected by the field nurse. Vaccination information was obtained from the vaccination card during enrollment, or from the vaccination registers at the health centers (with parental consent) if this was unavailable.

### Blood phenotyping

ABO blood typing was performed by hemagglutination test. In brief, three drops of blood were mixed separately with anti-A, anti-B and anti-AB monoclonal antibodies (Cypress Diagnostics, Langdorp, Belgium). ABO blood phenotype was assigned based on visual examination of hemagglutination with a given antibody.

### Lewis and secretor phenotyping

LeA, LeB and secretor antigens were detected using an in-house saliva-based ELISA as previously described^12^. The cut-off value was defined as a 2-fold change in absorbance value compared with previously characterized control saliva obtained from LeA-B- and non-secretor phenotype individuals.

### DNA purification

DNA was extracted from 200 µl buffy coat suspension using a QIAamp® DNA Blood Minikit (Qiagen, Hilden, Germany) and stored −20 °C.

### FUT2 genotyping

To confirm the secretor phenotyping results, all samples were analyzed for the *FUT2* G428A (rs601338) nonsense single nucleotide polymorphism (SNP) using the TaqMan® SNP Genotyping Assay (Applied Biosystems, Carlsbad, CA, USA). DNA identified as homozygous (SeSe) and heterozygous (Sese^428^) for the G allele (Se), and the mutant A allele (non-secretor, se^428^se^428^) by pyrosequencing were used as controls^18^. At least two non-template controls were used in each assay.

### FUT3 genotyping

To confirm the Lewis phenotyping results, a subset of 28 DNA samples from LeA (n = 1), LeB (n = 8) and LeA-B- (n = 19) children were selected for *FUT3* genotyping. Four SNPs [59 T > G (rs28362459), 202 T > C (rs812936), 314 C > T (rs778986), and 508 G > A (rs3745635)] were investigated by Sanger sequencing as previously described^12^. Another common SNP [1067 T > A (rs3894326)] was determined by TaqMan® SNP Genotyping (Applied Biosystems). These SNPs were selected based on a previous report of their predominant association with the LeA-B- phenotype in Nicaragua^18^.

### Rotavirus IgA titers in plasma and breast-milk

Pre- and post-vaccination (first dose) plasma rotavirus-specific IgA titers were determined by ELISA using a modification of the method described by Bernstein *et al*.^45,46^. Briefly, 96-well microtiter plates (Greiner Bio-One, Kremsmünster, Austria) were coated with guinea pig anti-rotavirus antibody (SBL, Stockholm, Sweden). After blocking, either RV1 or RV5 (1:100) was added and incubated at 37 °C for 1 h. Serially double diluted plasma (100 µl; 1:20–1:640 for RV1 or 1:50–1:1,600 for RV5) was added and incubated at 37 °C for 1 h. Horseradish peroxidase-conjugated goat anti-human IgA (P0216; Dako, Glostrup, Denmark) and 1-Step™ Ultra TMB-ELISA Substrate (Thermo Fisher Scientific, Stockholm, Sweden) were used as the detection system. The titer was defined as the reciprocal of the highest plasma dilution having OD_450_ ≥ 0.100. Plasma IgA titers of ≥80 or ≥100 in the RV1 or RV5 cohorts, respectively, were defined as rotavirus-specific IgA seropositive and a 4-fold increase from pre- to post-vaccination was defined as seroconversion. PBS was included for background monitoring. Breast-milk rotavirus-specific IgA titers were determined following the same procedure.

### Calprotectin assay

Fecal calprotectin levels were determined in a subset of children as a marker of intestinal inflammation at the time of vaccination (RV1 n = 32; RV5 n = 60). In brief, calprotectin was extracted from fecal samples (20 mg) and mixed with 1 ml extraction buffer (0.1 M Tris, 0.15 M NaCl, 1 M urea, 10 mM CaCl_2_, 0.1 M citric acid and 5 g/L BSA). After vortexing, stool suspensions were filtered (pore size, 450 µm) and shaken (7,000 rpm, 20 min, 4 °C) (VWR, S-500, Hampton, NH, USA). After centrifugation (10,000 rpm, 20 min, 4 °C), the filtrates were stored at −70 °C. Calprotectin concentration was determined using a commercial ELISA kit (Hycult Biotech, the Netherlands) following the manufacturer’s instructions.

### Statistical analysis

Statistical analyses were performed in SPSS 14.0 and GraphPad Prism version 5.00 (San Diego California USA).The rate of seroconversion was calculated for each variable category. Possible associations between seroconversion and HBGAs were estimated using chi square tests and odds ratios (OR) with 95% confidence intervals (CI). *P* < 0.05 was considered to indicate statistical significance. For each variable, the category with the highest number of subjects was used as a reference group for OR calculation. For each HBGA phenotype, geometric mean titers (GMT) pre- and post-vaccination were calculated to define fold-increase in IgA levels and Wilcoxon Signed Ranks Test was used to determine significant differences between IgA titers pre- and post-vaccination.
